# The development of next-generation energy storage: an interview with Zaiping Guo

**DOI:** 10.1093/nsr/nwaf223

**Published:** 2025-05-29

**Authors:** Yuxiang Hu

**Affiliations:** State Key Laboratory of Materials Low-Carbon Recycling, College of Materials Science and Engineering, Beijing University of Technology, China

## Abstract

Energy storage systems have been attracting ever-increasing interest in recent decades, especially metal-ion batteries. As the predominant electrochemical energy storage technology, lithium-ion batteries still encounter critical challenges when deployed in various applications, especially for grid-scale energy storage, including inherent safety concerns, resource scarcity and high lifecycle costs across the production and recycling processes. These limitations significantly hinder their capacity to meet the exponentially growing demand for energy storage solutions. Consequently, there exists an urgent imperative to develop innovative energy storage systems that synergistically integrate enhanced safety profiles, cost-effectiveness and superior electrochemical performance. Such technological advancements are crucial for enabling next-generation energy storage and advancing global carbon neutrality objectives. How can we address existing issues and develop the post-lithium-ion-batteries for future society?

NSR conducted an in-depth discussion with Prof. Zaiping Guo, who is a Fellow of the Australian Academy of Science, Fellow of the Australian Academy of Technological Sciences and Engineering, and an ARC Laureate Professor at the School of Chemical Engineering, The University of Adelaide. Her pioneering work focuses on advanced electrode architectures and electrolyte engineering for various battery systems.

## REDEFINING THE FUTURE: FOUNDATIONS AND VISION OF POST-LITHIUM-ION BATTERIES

***NSR*:** What are post-lithium-ion batteries (post-LIBs)? What is the relationship between the development of next-generation batteries and current lithium-ion batteries (LIBs)?

***Guo*:** Post-LIBs represent the advanced energy storage technologies developed to address the limitations inherent in traditional LIBs. These limitations include safety concerns, resource scarcity and high costs. Post-LIBs utilize alternative materials and chemistries to enhance performance and sustainability.

Post-LIBs constitute emerging technologies characterized by: alternative charge carriers (Na⁺, K⁺, Zn²⁺, Al³⁺, Mg²⁺, etc.); novel electrolyte formulations (aqueous, solid-state, ionic liquid, etc.); and sustainable material supply chains. For instance, aqueous batteries, which use water-based electrolytes, offer enhanced safety due to their non-flammability. Other non-aqueous batteries, such as sodium-ion, flow, magnesium-ion and aluminum-based batteries, also present advantages in safety and resource availability over LIBs.

The development of these batteries is closely related to current lithium-ion technologies, as they build upon existing knowledge while addressing the shortcomings of lithium-ion systems. By leveraging the foundational principles of lithium-ion technology, researchers aim to create batteries that are not only more efficient and cost-effective but also more sustainable and safer for a wide range of applications.

**Figure:**
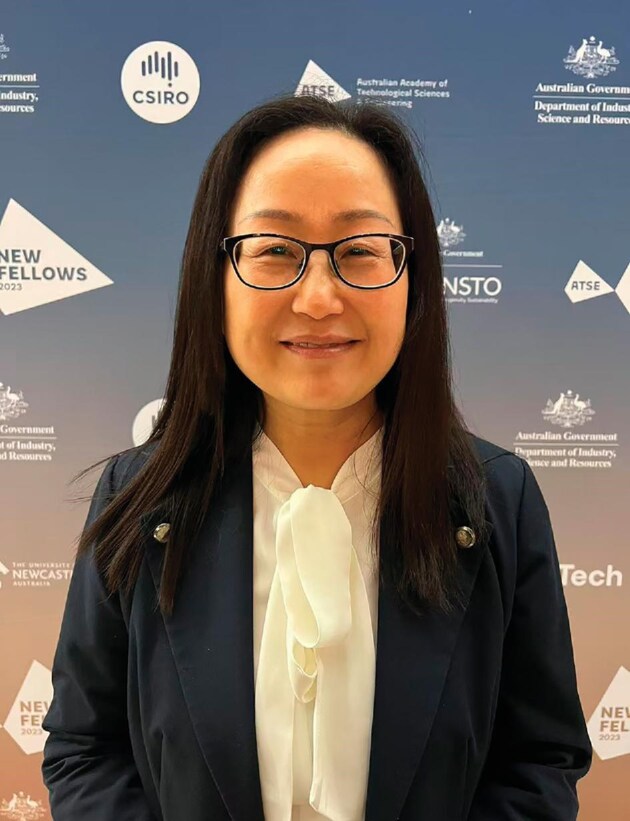
Prof. Zaiping Guo, a pioneer in next-generation energy storage research. *(Courtesy of Prof. Zaiping Guo)*

Post-lithium-ion batteries represent the advanced energy storage technologies developed to address the limitations inherent in traditional lithium-ion batteries.

—Zaiping Guo

## TECHNICAL CHALLENGES AND STRATEGIC BREAKTHROUGHS: BALANCING PERFORMANCE, SAFETY AND RECYCLING

***NSR*:** What are the key challenges in developing post-LIBs, and how can these challenges be addressed? How to balance the contradiction between high energy density and safety?

***Guo*:** Developing post-LIBs involves several key challenges. Achieving high energy density and long cycle life is crucial for ensuring that these batteries can compete with existing technologies. Safety is another significant concern; many alternative chemistries may introduce new safety risks that need to be carefully managed. Resource availability is also a critical issue; sourcing materials that are both abundant and environmentally friendly is essential for the sustainability of these technologies. Advances in solid-state electrolytes, electrode materials design and hybrid systems (combining different technologies) could offer potential solutions.

Balancing high energy density with safety requires innovative and multi-faceted approaches, such as designing safer electrolytes, improving battery management systems, using protective coatings and materials that prevent dendrite growth and improve the structural integrity of the battery, and engineering hybrid designs. For example, aqueous zinc/aluminum-based batteries present advantages in safety and resource availability over LIBs. These batteries utilize water-based electrolytes and abundant materials like zinc/aluminum, which are less prone to thermal runaway and are more readily available.

***NSR*:** Most post-LIBs employ a different electrochemical system from LIBs, such as a high-charge-density active-ion system. So, how to fundamentally explore the suitable electrode materials with high performance?

***Guo*:** Post-LIBs often utilize ions that are larger or have different charge densities compared to lithium; selecting electrode materials that can accommodate these differences is critical. These materials must exhibit high conductivity and stability while allowing efficient ion diffusion and supporting processes such as intercalation/de-intercalation or redox reactions. To achieve high battery performance, electrode materials should not only offer high theoretical capacity but also possess stable structures with minimal degradation during cycling. Additionally, the selected materials need to be abundant, environmentally friendly and cost-effective to enable large-scale production. Experts have developed various strategies to optimize electrode materials for high-charge-density systems, including defect construction, the use of high-entropy materials, and methods to enhance conductivity. In parallel, electrolyte systems for post-LIBs differ from those of LIBs, resulting in the development of novel strategies to improve overall performance. Furthermore, interface engineering techniques are being explored to enhance cycling stability and promote efficient ion diffusion, thereby improving the overall performance of post-LIBs.

***NSR*:** Battery recycling is a key aspect of sustainable development. What challenges do current LIBs and post-LIBs face in terms of recycling technologies?

***Guo*:** Battery recycling is essential for sustainable development, but both current LIBs and post-LIBs face challenges in this area. LIBs often contain a variety of metals, including lithium, cobalt, nickel and manganese in various chemical compositions. The diversity of these materials makes it challenging to develop a one-size-fits-all recycling process. The extraction of these metals requires advanced technologies that can efficiently separate and purify each element without compromising the quality of the materials. As the battery market evolves with the introduction of alternative technologies (e.g. sodium-ion, solid-state, zinc-ion etc.), the materials and compositions used in these batteries will continue to vary. This adds complexity to the development of universal recycling processes. Some post-LIBs may offer easier recycling processes due to their simpler chemistries and the abundance of materials like zinc, magnesium and aluminum, which are widely available and can be recycled efficiently, reducing the environmental impact associated with battery disposal. Advancements in recycling processes, infrastructure and safety are necessary to ensure that both current and future battery technologies can be recycled sustainably.

Balancing high energy density with safety requires innovative and multifaceted approaches, such as designing safer electrolytes, improving battery management systems, using protective coatings and materials that prevent dendrite growth and improve the structural integrity of the battery, and engineering hybrid designs.

—Zaiping Guo

## DRIVING COMMERCIALIZATION AND INCLUSION: FROM LAB TO MARKET AND BEYOND

***NSR*:** What technological breakthroughs do you think could drive the commercialization of post-LIBs?

***Guo*:** Technological breakthroughs that could drive the commercialization of post-LIBs include the development of high-performance electrode materials, advanced electrolytes and scalable manufacturing processes. For instance, creating electrode materials with enhanced capacity and stability could significantly improve battery performance. Similarly, developing electrolytes that are both efficient and safe can enhance the overall safety and efficiency of the batteries. Additionally, establishing manufacturing processes that are cost-effective and scalable is essential for enabling the widespread market adoption of these advanced battery technologies.

***NSR*:** As a female researcher, what advice do you have for encouraging more women to engage in scientific research?

***Guo*:** Encouraging more women to engage in scientific research requires the creation of an inclusive and supportive environment. Mentorship plays a crucial role; experienced researchers can provide guidance, support and inspiration to young women entering the field. Equal opportunities are also vital; ensuring that women have the same access to resources, funding and career advancement as their male counterparts is essential for fostering a diverse scientific community.

Recognition of achievements is another important aspect. Celebrating the contributions of female scientists can serve as

Encouraging more women to engage in scientific research requires the creation of an inclusive and supportive environment.

—Zaiping Guo

inspiration and demonstrate that success is attainable. Additionally, addressing unconscious biases and promoting policies that support work–life balance can help retain women in the scientific workforce. By implementing these strategies, we can encourage more women to pursue and really shine in scientific research, leading to a more diverse and innovative scientific community.

